# Supplementary material for: Automatic classification of mice vocalizations using Machine Learning techniques and Convolutional Neural Networks
Source: PLoS One. 2021 Jan 19;16(1):e0244636. doi: 10.1371/journal.pone.0244636 (PMC7815145; doi:10.1371/journal.pone.0244636)
Supplement: Attachment — Submitted filename: Response to Reviewers.docx [file pone.0244636.s001.docx]

We would like to take this opportunity to sincerely thank the Reviewers for their valuable comments. We have carefully revised our manuscript, taking all the comments and suggestions into consideration.

*General comments*

*__________________________________________________________________________*

First, we list the main efforts we have spent to enhance the experimental phase, in accordance with the Reviewers’ recommendations:

1) Expansion of the statistical analysis of the results;
2) Rerun of the experiments after:
   a. Extending the standard feature space, for feature-based learning algorithms;
   b. Improving the Convolutional Neural Network (CNN) architecture by also including pertinent techniques for overfitting prevention;
3) Implementation of two new sets of experiments, namely:
   a. Classification after splitting the dataset into the two genotype-based datasets;
   b. Classification performed by removing one class.

The new experiments have led to an overall improvement in the classification performance, especially when the CNN is used. Of course, the updated results are reported in the new version of the paper.

We change the title of the manuscript in “Automatic classification of mice vocalizations using Machine Learning techniques and Convolutional Neural Networks”, suggesting the importance of use of the Convolutional Neural Networks in our paper.

Beyond the experiment aspect, another point that has importantly contributed to the paper revision is related to the work that was suggested by both Reviewers, namely:

*Vogel, A.P., Tsanas, A. & Scattoni, M.L. Quantifying ultrasonic mouse vocalizations using acoustic analysis in a supervised statistical machine learning framework. Sci Rep 9, 8100 (2019).*

Accordingly, in the revised paper we discuss the main points that differentiate our work from the above cited paper, which has been properly referenced therein. Furthermore, we want to point out that now we have shifted our attention to the classification method based on CNN architecture, since in the 2019 *Scientific Reports* paper some traditional machine learning (ML) algorithms were already tested. Consequently, the discussion on the ML techniques results to be more limited with respect to the first submission. This change of focus is also motivated by the fact that the new experiments, carried out in the revision stage, have shown notably superior performance when using CNN as stated above. In addition, as pointed out in the *3) b.* item above listed, in order to make the comparison as fair as possible, we have also implemented a further set of experiments, in which the classification has been performed by removing the class *harmonic*, as done in Vogel’s paper.

As a final note, we also mention that we will make both the dataset and the implementation code publicly available.

In the following, we reply item by item to the specific Reviewers’ comments.

*Response to Reviewer #1*

*__________________________________________________________________________*

**Comment 1 (major)**

- ***For statistical confidence, the cross-validation technique (e.g. 10-fold) should be applied for model assessment and the results should be shown together with their confidence intervals.***

**Response:** Following this valuable suggestion, we have applied 10-fold cross-validation for model assessment. In the revised paper, we have reported the results as mean ± standard deviation.

*__________________________________________________________________________*

**Comment 2 (major) and Comment 3 (minor)**

- ***The feature set used for RF, SVM and non-convolutional ANN input doesn’t seem to be adequate for the desired task. The authors should extend the feature set and/or use entire frequency envelope data as the input.***
- ***The report of* Vogel et al, 2019 ‘Quantifying ultrasonic mouse vocalization using acoustic analysis in a supervised statistical machine learning framework’ *solving similar task should be mentioned.***

**Response:** The problem expressed in Comment 2 (major) also implicitly deals with Comment3 (minor), therefore we would like to address both the comments in the same answer.

We agree with the Reviewer that the results obtained by using the feature set described in the original paper suggest a perfectible ability to generalize the investigated machine learning models. As a matter of fact, we have extended the original feature set by including other features extracted from the spectrograms.

The new features selection has been based from the paper suggested by the Reviewer (Voget et al., 2019). We have carefully read that report, in which the authors propose an optimal feature subset of 8 acoustic measures for USV classification. They are duration, quart 50 (start), peak freq (stddeventire), peaktopeak, max freq (stddeventire), peak freq (start), peak freq (end) and quart 75 (start). Since 4 of these 8 features are already included in the original feature set, namely, duration, peak freq (start), peak freq (end) and peak freq (stddeventire), we have just added the remaining 4 features, leading to a final feature space formed by 20 features (against the original one composed by 16 features).

The new performances outperform the ones referred to the submitted paper, even if the strongest improvement has been obtained by enhancing the CNN architecture, as pointed out in the next response.

*______________________________________________________________________________*

**Comment 3 (major) and Comment 2 (minor)**

- ***The architecture of ANNs should employ widely used techniques for overfitting prevention: batch normalization/dropout. It would be good to employ data augmentation (e.g. random shifts and crops along temporal axis) to help the CNN perform better. The batch size also can be increased to help improve the performance and/or convergence speed.***
- ***ANN architecture should be described more thoroughly: convolutional layers kernel sizes, strides, dropout/batch normalization usage, stochastic gradient descent optimizer used, weight initialization strategy.***

**Response:** Regarding the issues detailed in Comment 3 (major), it is undoubtable that overfitting seems to afflict the proposed Convolutional Neural Network (CNN). The previously reported accuracy in training and testing phases are 87.0% and 58.3%, respectively, indicating that our model had some problem to generalize new examples not included in the training dataset.

Agreeing with the reviewer suggestions, some techniques for overfitting prevention have been implemented, listed in the following:

a. Regularization, i.e., dropout;
b. Batch normalization;
c. Testing different batch sizes;
d. Data augmentation.

In order to properly illustrate how we have implemented the just mentioned techniques, it can be helpful to start by describing the CNN architecture, that in turn also answers to Comment 2 (minor).

First of all, we want to point out that substantial changes have been applied in the model architecture: indeed, the introduction of methods to prevent overfitting came with an increase of the architecture complexity.

The final model is composed by 5 convolutional layers constituted by 32, 64, 64, 128 and 128 filters, respectively. The convolutional layers kernel sizes are 7x7 and 5x5 in the first two layers, and 3x3 in the successive three. The stride is set to 1 for each layer.

A max-pooling layer is inserted after each convolutional layer. The pooling size is fixed to 3x3, while the stride is set to 2x2. The tensor is then flattened, and two fully connected layers are inserted, the first of size 1024 with ReLU activation function, and the second of size 10 with Softmax activation function. We mention that the Adam optimization algorithm has been used as stochastic gradient descent optimizer, and that the uniform distribution has been adopted to initialize the network weights. The description of the final CNN architecture has been thoroughly illustrated in the new version of the paper.

To prevent overfitting, the strategies that we referred above as a., b., c., and d., have been tested. Dropout, i.e. a., deletes a random sample of the activations (namely, makes them zero) during training. Of course, dropout causes information loss, in particular, losing something in the first layer propagates that loss to the whole network. We have designed several configurations, i.e., we have located dropout layers in different positions and tested a range of rates (the fraction of input units to drop), from 0.2 to 0.8. The batch normalization technique (b.) has been similarly evaluated: it helps to coordinate the update of multiple layers in the model. We have experimented batch normalization layers both before and after the activation functions, and in parallel we have considered different batch sizes (c.) in the training phase.

In the end, after this thorough hyperparameters optimization/tuning, the best model resulted the one with a dropout layer after each convolutional/max-pooling layer, except the first one, setting the rates to 0.2, 0.3, 0.4 and 0.5. Then, a further dropout layer was added after the first fully-connected layer (rate equals to 0.5), and finally batch normalization was included before the Softmax activation function of the last fully-connected layer. The batch size finally adopted is 32.

By applying all of these adjustments, the performance of the final CNN architecture has notably improved: indeed, the test accuracy has been enhanced from 58.3% to 78.8%.

Here is a final note on data augmentation (d.): given the characteristics of the spectrograms, some image transformations cannot be analyzed, since they would invalidate the ground-truth classification. For example, rotation or horizontal/vertical flipping could lead the network to confuse the upward class with the downward class, and vice-versa. For this reason, and also as suggested by the reviewer, we have increased the dataset by shifting and cropping the spectrogram images at random: however, the experiments have shown that data augmentation does not significantly improve the performance of the model. As with the other points, the discussion on overfitting prevention has been properly argued in the revised paper.

*______________________________________________________________________________*

**Comment 1 (minor)**

- ***SVM OVA approach requires confidence estimation. Which confidence estimation approach was used?***

**Response:** For SVM OVA method, we set a maximum confidence strategy, similar to the weighted voting strategy from OVO systems. So, the output class is taken from the classifier with the largest positive answer. We have reported this detail in the revised version of the paper.

*______________________________________________________________________________*

**Comment 4 (minor)**

- ***Strain data is not used in the analysis anyhow. It would be interesting to compare the accuracies of two strains' shape prediction results taken separately.***

**Response:** We have taken into consideration this valuable suggestion. As a matter of fact, in the revised paper we have included a new set of experiments, by testing the classification methods on the genotype-based datasets taken separately.

*______________________________________________________________________________*

**Typos**

- ***96 'can not applies'***
- ***124 'postnatal (PND)'***

**Response:** We have corrected the typos indicated by the Reviewer.

*Response to Reviewer #2*

*__________________________________________________________________________*

**Comment 1**

- ***The original creator of these particular USV categories, Maria Luisa Scattoni, has already published a paper using support vector machines (SVM) and random forests (RF) to categories USVs. They achieved 85% recall. What more does this paper add?***

**Vogel, A.P., Tsanas, A. & Scattoni, M.L. Quantifying ultrasonic mouse vocalizations using acoustic analysis in a supervised statistical machine learning framework. Sci Rep 9, 8100 (2019).**

**Response:** We have meticulously read the suggested report, and we agree that it is important to shed light on the improvements led by our work with respect to Vogel’s one. We want to focus here on the two main breakthroughs that in our opinion are worthy to be pointed out.

First, we made a significant effort to build our dataset. We performed our experiments on 48699 samples, which is a much more sizable dataset than the 225 samples evaluated in the suggested paper. It is well-known that the bigger the sample size is, the more accurate the research results are. That is because larger dataset cardinality allows to better determine the average values of data and reduce potential errors from testing a small number of possible outliers. As a consequence, the statistical analysis becomes more accurate and the margin of error smaller. Given these crucial considerations, we believe that our results benefit from a very effective statistical soundness, and thus that they may represent a further valuable benchmark for future works in this research field. Of course, the dataset will be made publicly available.

The second aspect we want to mention is that in our work we explored the classification ability of a Convolutional Neural Network (CNN), extending the analysis beyond traditional machine learning algorithms, such as Support Vector Machine and Random Forest, employed in Vogel’s paper. To the best of the authors’ knowledge, this work is the first to propose a deep learning architecture aimed to classify the ten categories originally introduced in [17]. Furthermore, the CNN model has been notably modified during the revision phase. Accordingly, we have included all of these modifications in the revised paper. In particular, if compared with the original proposed architecture, we have changed the network topology by inserting more convolutional layers and increased the number of the filters composing them, in parallel to have investigated some techniques for overfitting prevention, such as regularization (drop out) and batch normalization (please find more details in the new version of the paper). As a consequence of such improvements, the performance of the final CNN model now achieves an accuracy of 78% on the test dataset, strongly improving the results reported in the original paper (where the test accuracy was 58.3 %).

- ***Experimenter derived call categories are generally falling out of favor. Substantial new evidence suggests that USVs don’t categorize neatly into discrete groups, including the data in this paper. SVMs/RFs are capable of much higher accuracy when the training data actually comes from discrete groups with clear separations. The “short” calls in the present manuscript are a well-defined group and are thus categorized accurately (>90%). But other calls like “complex” and “composite” are frequently miss-categorized. This likely isn’t a fault of the classifier. Rather, it captures the uncertainty within the human created training data.
  (Discrete vs Continuous Vocalizations)*
  Tim Sainburg, Marvin Thielk, Timothy Q Gentner (2019) Latent space visualization, characterization, and generation of diverse vocal communication signals. bioRxiv 870311.**

**Response:** We think that this different perspective is very interesting. We agree that automatic USVs classification into a fixed number of discrete groups is a challenging task, as also suggested by the paper cited in the comment. Indeed, the results reported in the originally submitted paper confirmed the difficulty of distinguishing particular classes, such as *complex* and *composite*.

Nevertheless, even if manual classification is an undeniably time-consuming activity and also vulnerable to personal interpretation, it can be performed within a more than reasonable margin of error, especially when the general shape of the spectrogram is analyzed.

Even acknowledging an intrinsic uncertainty in the human labelling of data, in our work we addressed the problem of designing efficient and accurate machine learning and deep learning models, aimed to automatically classify vocalizations starting from the audio tracks. The performance obtained from the new set of experiments suggests that crucial improvements have been implemented to the originally proposed models. Indeed, the new results show a lesser uncertainty in USVs categorization, indicating that well defined models can actually lead to significant performance even when discrete vocalizations classification is investigated.

This discussion has been inserted also in the revised paper, citing the suggested article.

*______________________________________________________________________________*

**Comment 2**

- ***Many methods are now available for un-biased call classification. These categories can then be validated through the behavioral/contextual usage of the calls. Call categories that are used identically during behavior can be collapsed. This method is more sophisticated and ethologically relevant than creating categories based on experimenter visual inspection.*
  Sangiamo, D.T., Warren, M.R. & Neunuebel, J.P. Ultrasonic signals associated with different types of social behavior of mice. Nat Neurosci 23, 411–422 (2020).**

**Response:** In the literature different methods of calls classification exist. We used a method based on experimenter visual inspection referring to papers of Dr. Scattoni cited in our references. This method permits to the operator to classify vocalizations based on features as frequency content, duration, amplitude and general shape. This classification provides a detailed USV characterization, but it says nothing about the meaning of the calls. Recently, new technologies have been developed such as a microphone array system and sound source localization method, to localize and assign USVs to individual mice during a social context (Heckman et al., 2017 doi:10.1038/s41598-017-02954-z; Warren et al., 2018 doi:10.1016/j.jneumeth.2017.12.013; Sangiamo et al., 2020 doi.org/10.1038/s41593-020-0584-z). This permits to associate distinct vocalizations categories with different types of murine social behavior giving important information to calls meaning. Unfortunately, we do not have these sophisticated instruments to associate behaviors to calls. In addition, we recorded vocalizations emitted by pups and not adults and in pups it is not possible to associate calls to behaviors (walk, fight, mutual circle… in a social context) as done by Sangiamo and colleagues because pups are still very young (first days after birth) and unable to move or perform more complex actions. They still have closed eyes. Finally, we agree with the Reviewer with the idea that it is very interesting to understand the meaning of mice calls and so, in the future, we would like to perform more sophisticated and ethologically relevant analysis on adult mice.

*______________________________________________________________________________*

**Comment 3**

- ***The training data, code, and finalized classifiers are the most valuable elements of this manuscript, but I did not see any indication that they will be distributed to the field. Without this, the manuscript just describes their internally used classifier with moderate accuracy.***

**Response:** We are in total agreement with the Reviewer. As a matter of fact, we will make both the dataset and the implementation code publicly available.
