# Supplementary material for: Automatic classification of mice vocalizations using Machine Learning techniques and Convolutional Neural Networks
Source: PLoS One. 2021 Jan 19;16(1):e0244636. doi: 10.1371/journal.pone.0244636 (PMC7815145; doi:10.1371/journal.pone.0244636)
Supplement: Attachment — Submitted filename: Response to Reviewers.docx [file pone.0244636.s002.docx]

We would like to take this opportunity to sincerely thank everyone involved in the review process. We are glad that the adjustments introduced in response to reviewers' comments process have been generally appreciated. The further changes suggested by the reviewer #1 are detailed below.

*Response to Reviewer #1*

*__________________________________________________________________________*

**Comment 1 (major)**

- ***The Random Forests, MP, Stacking NN classification performance results are completely omitted in the paper. SVM performance results are hard to find in the text. At the same time, both RF and SVM are mentioned in the abstract and multiple times throughout the text. The results of all classifiers mentioned in the paper should be presented in a table. If the resulting accuracy is especially bad and is not worth considering than it should be stated explicitly.
  Note that the results are compared with the results of RF classifier in Vogel et al, 2019 paper, so the omitting of the RF results looks strange.***

**Response:** As mentioned in the previous response process, in the first revised paper we had moved the focus from the “numerical features”-based classification techniques, both for distancing our work with respect to Vogel’s paper (as requested by both the reviewers), and also because the new results obtained by using the Convolutional Neural Networks had outperformed the others. Accordingly, we had considered not interesting and redundant the list of all the performance for all the investigated techniques, with that not for that intending to diminish the importance of studying those methods in our work.

However, we agree with the reviewer that a table containing the overview of all the results may benefit the completeness of the paper. As a matter of fact, in the new version of the paper, we have inserted such a table in the “Performance analysis” section. Nonetheless, to streamline the paper, we just report the performance related to the entire mice vocalizations dataset, since the strain-based performances result are similar to the ones obtained for the entire dataset, and so they do not add significant extra information.

Finally, the source code has been properly integrated as well.

*__________________________________________________________________________*

**Comment 2 (major)**

- ***'Data-preprocessing' paragraph. 'This operation prevents imbalanced dataset...' - usually, that is not the main reason to apply data augmentation when training CNN. To deal with imbalanced data one can just use weighted loss function, as you mention further in the paragraph. Data augmentation is used mostly to prevent overfitting,
  generating a potentially infinite, though maybe not diverse enough set of training samples.***

***From your description it is not clear which approach for data augmentation
is used for the best-performing CNN: statically generated examples with random crops and shifts, just to balance the source data set, or the on-the-fly generation of randomly altered samples during the training, together/without using loss function weighting? That should be stated explicitly.***

***Additionally, I think that is not necessary to explain that one should not rotate on flip images in that case. 'data augmentation' is a general term for generation of pseudo-diverse samples and that is quite obvious that one should not use the techniques applied for object photographs classification here...***

**Response:** Thanks for pointing this out. We take this space to better clarify how data augmentation has been used in our experiments, and we will report these considerations in the new version of the paper.

For the CNN design, we have tested data augmentation for both overfitting and imbalanced dataset prevention, separately. In the first case, an offline generation of new examples with random shifts and crops is applied over the entire training dataset; when using data augmentation for this aim, a properly weighted loss function is employed during the training in order to handle the imbalanced dataset problem too. In the second one, data augmentation is performed to balance the source dataset, and so by generating new samples just for the under-represented classes.

However, note that data augmentation has been tested in the experimental phase, but it has not been in the end used to obtain the final best-performing CNN model. Indeed, the experimental tests have revealed that just including dropout and batch normalization layers is the most efficient action for overfitting prevention (as reported in the “Classification via CNN” section). Furthermore, the experiments have also provided more uniform accuracy values across the classes when the loss function is properly weighted instead of repopulating the under-represented classes by implementing data augmentation. In conclusion, data augmentation has not been exploited for designing the most-performing CNN architecture.

As mentioned before, in the new version of the paper, we have better clarified all these aspects by partially re-writing the former section “Data pre-processing”, which is now titled “Class imbalance handling”. Therein, the reviewer will find that all his/her observations above have been properly considered.

*__________________________________________________________________________*

**Comment 3 (major)**

- ***'Proposed classification methods' paragraph: you briefly explain the principles of CNN and only after that you start explaining trivial theory of MP, talking about weights, activation functions and neurons. 'Multilayer perceptron' sub-section should go first, since it describes the most basic things on which CNN is based as well.
  Furthermore, I don't think Fig.2 is worth placing in the paper. That diagram is quite trivial, it occupies half of a page and is known since 70s. It can be found in any textbook about ANN basics.***

***Why not to use that space to depict the actual architecture of the CNN + ANNs you designed, maybe together with some trained kernel weights visualization or other data? For example, see the figures in our recently published work***

***Ivanenko et al, 2020, "Classifying sex and strain from mouse ultrasonic vocalizations using deep learning".***

**Response:** Thanks for the suggestion. We agree with the reviewer that the paper may be clearer by swapping the “Convolutional Neural Networks” and “Multilayer Perceptron” sub-sections.

In the revised version of the paper, we have modified the description of the multilayer perceptrons without the support of Fig. 2. In its place, there is a new figure depicting the entire architecture of the designed CNN; such a figure has been suitably added in “Classification via CNN” section.

*__________________________________________________________________________*

**Minor (Abstract)**

1) ***tested some supervised ... -> tested some other supervised ...***
2) ***extracted by the spectrograms -> extracted from the spectrograms***

**Response:** We have revised both the sentences as in 1) and 2).

*__________________________________________________________________________*

**Minor (Introduction)**

1) ***"... on such a fixed repertoire of calls typologies". I think this should be rephrased. May be "on a predefined set of call types"?***
2) ***Please cite our paper Ivanenko et al, 2020, "Classifying sex and strain from mouse ultrasonic vocalizations using deep learning", PLOS CB in the introduction. Though we don't use Scattoni classical vocalization types there, we also classify vocalizations using CNNs basing on their spectrogram shape (ascending, descending, number of jumps, peaks, complexity etc), thus implementing the 'top-down' approach you mentioned.***
3) ***'Simulation results' - I think the word 'simulation' is misleading here.***

**Response:** We have revised both the sentences as in 1) and 3). We have also carefully read the paper suggested in 2) and cited it in the introduction while mentioning the top-down approach.

*__________________________________________________________________________*

**Minor (USV Recording and analysis)**

1) ***"postnatal (PND) 6, 8 ..." -> "postnatal day (PND) 6, 8..."***
2) ***Threshold and hold time parameter values used in Avisoft for vocalization extraction should be provided here.***

**Response:** We have revised the sentences as in 1) and inserted the requested parameters in the appropriate section as suggested in 2).

*__________________________________________________________________________*

**Minor (Description of the experiments)**

1) ***'the first step consisted into' -> 'the first step consisted of'***
2) ***'giving this way a statistical soundness' -> 'ensuring the statistical soundness'?***

**Response:** We have revised both the sentences as in 1) and 2).

*__________________________________________________________________________*

**Minor (Feature extraction section)**

1) ***identified by the color...' -> 'identified by the brightness...'***

**Response:** We have revised the sentences as in 1).

*__________________________________________________________________________*

**Minor (Support Vector Machines)**

1) ***'Maximum confidence strategy was used...' - how the confidence was estimated? SVM provides binary result out of the box, the actual confidence assessment approaches vary.***

**Response:** The confidence is directly given by the decision function, which is the signed distance between the tested sample and the separating hyperplane. More precisely, the decision function is computed as $\sum_{i\in SV} y_{i}\alpha_{i}K\left( x_{i},x \right)+b$, where

- $x$ is the sample to predict;
- $x_{i}$ are the support vectors ($SV$) that construct the hyperplane;
- $b$ is the intercept;
- $\alpha_{i}$ are the dual coefficients computed in the SVM optimization problem;
- $y_{i}$ are the binary labels corresponding to $x_{i}$;
- $K\left( x_{i},x \right)=\varphi\left( x_{i} \right)^{T}\varphi\left( x \right)$ is the employed kernel.

In the OVA-strategy, we generate 10 classifiers: the first separates *class 1* from the remaining ones, the second does the same for *class 2*, and so on for all 10 classes. The decision function is computed for each class as reported in the previous equation. The classifier that returns the largest positive value identifies the winner class.
